# Participant experiences receiving acupuncture for acute musculoskeletal pain in an emergency department: A qualitative evaluation

**DOI:** 10.1371/journal.pone.0318345

**Published:** 2025-02-12

**Authors:** Anna Tupetz, Madison Frazier, Amy O’Regan, Mitchell Knisely, Olivia TumSuden, Erica Walker, Charlotte Sununu, Oliver Glass, Ann Miller-Maxwell, Catherine A. Staton, Stephanie A. Eucker

**Affiliations:** 1 Department of Emergency Medicine, Duke University School of Medicine, Durham, North Carolina, United States of America; 2 Global Emergency Medicine Innovation and Implementation Research Center, Duke Global Health Institute, Duke University, Durham, North Carolina, United States of America; 3 Department of Population Health Sciences, Duke University School of Medicine, Durham, North Carolina, United States of America; 4 Duke University School of Nursing, Durham, North Carolina, United States of America; 5 Department of Medicine, Duke University School of Medicine, Durham, North Carolina, United States of America; 6 Durham Veterans Affairs Hospital, Durham, North Carolina, United States of America; China Medical University, TAIWAN

## Abstract

**Objective:**

Acupuncture is an evidence-based pain treatment in clinic settings, but its optimal delivery has not been established in emergency departments (EDs). As part of an adaptive pragmatic randomized controlled trial of ED acupuncture for acute musculoskeletal pain (NCT04290741), we embedded a qualitative evaluation of acupuncture treatment acceptability and suggestions for improvement from study participants receiving acupuncture in the ED.

**Methods:**

Semi-structured interviews conducted remotely evaluated factors impacting patients’ perspectives, willingness to participate in, and experiences with ED acupuncture. The codebook was iteratively developed, and recruitment and analysis continued until information saturation was reached.

**Results:**

Twenty-eight participants receiving ED acupuncture between February 2020-March 2021 were interviewed, with median age 44 years, 46.4% female, and 61% having never previously received acupuncture. Overall, ED patients with acute musculoskeletal pain expressed interest in acupuncture and reported an overall positive experience. Most reported acupuncture met their expectations for pain improvement, and many reported additional improvements in stress, anxiety, and sleep quality. Participants with a positive experience were more likely to recommend acupuncture to others. Key positive aspects included open communication with compassionate and knowledgeable acupuncturists. Participants found the ED setting acceptable and convenient for receiving acupuncture. Furthermore, participants provided actionable feedback like addressing fear of needles to improve the ED acupuncture experience.

**Conclusions:**

In conclusion, ED patients with acute musculoskeletal pain were interested in and had positive experiences with acupuncture treatment for pain and found the ED setting acceptable and convenient. Participant feedback can be used to improve acupuncture treatment in the ED.

## Introduction

### Background

Acute musculoskeletal pain, including pain in the neck, back and extremities, is among the most common reasons people seek emergency department (ED) care [1–4]. Pharmacological management, including opioid and non-opioid pain medications, is the primary treatment modality for acute musculoskeletal pain in this setting. Unfortunately, only approximately 12% of patients with acute musculoskeletal pain in the ED receive adequate pain management, and less than 20% receive clinically relevant pain relief [5]. Few non-pharmacological treatments are available in the ED to treat pain or reduce the need for medications with high side-effect profiles such as opioids. Acupuncture is an evidence-based, safe and cost-effective treatment for management of acute and chronic pain in clinic settings and has much potential to mitigate the effects of undermanaged acute pain in the ED [6]. Very few studies have explored the effects and implementation of acupuncture for treating pain in this setting utilizing patient-centered methods.

### Importance

Patient perceptions of acupuncture as part of usual care, outside the research context, are largely positive, and patients report quality of life benefits associated with pain relief [7,8]. However, these patients may already have positive perceptions of acupuncture prior to initiating treatment [9]. Participants recruited from an ED, particularly those unfamiliar with acupuncture, may have very different perceptions and expectations of acupuncture that should be taken into account when implementing an intervention in this context [10]. Nesting qualitative studies within RCTs is integral to contextualizing quantitative findings and improving trial conduct based on participant feedback [11]. Some prior interventional acupuncture studies have published on qualitative aims, but the application of this method in the ED setting is novel [12–14].

### Goals of This Investigation

We conducted qualitative interviews with a sample of patients enrolled in the initial pilot phase of the parent study [15,16]. We aimed to understand the acceptability and experiences of patients receiving acupuncture for acute musculoskeletal pain in an emergency setting, and identify ways to improve those experiences.

## Methods

### Ethics statement

The Institutional Review Board approved the study protocol (Pro00104140). All participants initially provided written electronic informed consent to participate in the parent clinical trial of this study that included the consent to participate in the qualitative study portion. When contacted to participate in the qualitative data collection, data collectors reviewed the interview process with the participants again and answered any remaining questions and receive verbal consent, prior to starting the interview. The participants were compensated for their time.

### Study design

This work was ancillary to and informed the conduct of a concurrent randomized clinical trial (NCT04290741) aiming to determine the effectiveness of acupuncture for ED management of acute musculoskeletal pain [15,16]. As part of the initial pilot phase of the trial, we engaged patients on the design and tailoring of the ED acupuncture intervention in a patient-centered way. We performed a qualitative study embedded in the parent trial to understand experiences, acceptability, and suggestions for improvement from study participants receiving acupuncture in the ED, following inductive content analysis approaches [17]. We followed the COnsolidated criteria for REporting Qualitative research (COREQ) guidelines in the reporting of this study [18].

### Research team roles and reflexivity

The interviewers (OCT, EW) and research assistants (MF, CS) completed two interactive training workshops on qualitative research, interviewing techniques and introduction to qualitative data analysis, with the main data analyst (AT) overseeing the qualitative study component. Interviews were performed by two research coordinators (OCT, EW) who were involved with participant recruitment, enrollment and data collection in the parent ED acupuncture trial. Therefore, interview participants may have met the interviewer previously when they were enrolled in the parent study. Interviews were coded by a clinical research coordinator and research assistant (MF, CS) who did not have any interactions with study participants. The lead data analyst (AT) supervised the coding and resolved disagreements between coders. Additional research team characteristics are presented in S1. (S1 Table).

### Study setting

This qualitative study was embedded within a pragmatic randomized controlled trial of acupuncture for acute musculoskeletal pain in the ED. Inclusion criteria for the parent study were adult ED patients (age 18 years or older) presenting with acute musculoskeletal pain of the back, neck, and/or extremities, with onset of the current pain episode within the last 7 days. Participants in the acupuncture arms received either auricular acupuncture (based on the battlefield acupuncture protocol) or peripheral acupuncture (with needles applied to the head, neck and/or extremities but not the torso) during their ED visit, and those in the control arm did not receive acupuncture [15,16]. Participants in the acupuncture arms also received access to free acupuncture treatments in clinic twice a week for one month following ED discharge. All trial participants also received usual ED care for pain by a blinded ED provider, completed surveys while in the ED, and were asked to complete 2-week and 1-month follow-up surveys.

### Participant selection and recruitment process

Participants enrolled in the parent study between February 19^th^, 2020 and March 19^th^, 2021 were invited to participate in this qualitative component after completion of their 1-month follow-up survey for the parent study, or after 3 phone contact attempts had been made by the study team to participants who did not complete the survey at 1-month. Invitations containing study details and consent information to participate in the qualitative interviews were sent to potential participants by email, and non-responders received two additional phone call recruitment attempts. Initial interview invitations used a convenience sampling strategy and were later changed to a purposive sampling strategy in the final phase of data collection after interim analyses described below, to ensure the qualitative sample population represented the demographics of the overall clinical trial study population.

### Interview and data collection procedures

The study team collaboratively developed a semi-structured interview guide that was updated after two interim analyses and discussions within the team, as described below. The interview questions specific to the acupuncture treatment in the Emergency Department, covered the expectations of patients prior to receiving acupuncture, the experiences of receiving acupuncture in the Emergency Department, experiences with the acupuncturist, perceived necessary information and education about acupuncture treatment to be provided prior to the treatment and recommendations and suggestions to improve the treatment experience. Between May 7^th^ 2020 and May 6^th^ 2021, remote interviews were conducted and audio-recorded by two qualitatively-trained research coordinators (OCT and ERW) via Zoom, with or without video interactions based on participant preference. Only the interviewers and participants were present during data collection and no repeat interviews were carried out. Data was transcribed through an external transcription service, reviewed by the research team, and preliminarily analyzed, with adaptations made at interim analyses prior to subsequent interviews. A complete analysis was performed after the completion of all interviews using the final version of the codebook.

### Interim analyses

Initial invitations were sent from March 2020 to October 2020 to all participants in only the acupuncture treatment arms (no control group participants). The first interim analysis (October 2020) based on the first 4 interviews included the inductive development of a preliminary codebook and content memos. Based on team discussion, only minor wording adjustments were made to the interview guide.

A second interim analysis (February 2021) was performed after 22 total interviews had been conducted and 8 of these interviews had been coded. The preliminary analysis as well as observations from the interviewers regarding whether they perceived any new information to be emerging during the interviews were discussed among the team. Based on the discussion, it was determined that the data was approaching information saturation regarding the acupuncture treatment experience, meaning no new information arose from the patients’ recount of their experiences [19,20].

In addition, we compared the demographics of the qualitative study participants with the demographics of the overall parent trial population and identified imbalances with some underrepresentation of male and Black individuals. Therefore, the sampling strategy was adjusted to increase their recruitment to better reflect the demographic composition of the study population, ensuring broader representation in this study. An additional 6 interviews with participants in the acupuncture arms were performed, and data collection was completed in May 2021. The final sample size was deemed sufficient to reach information saturation, aligning with qualitative research standards [19].

### Data analysis

Qualitative data was analyzed using inductive content analysis, to identify themes related to ED acupuncture treatment experiences of participants [17]. The codebook was iteratively developed and adapted by the study team throughout the data collection and coding process and as part of the interim analyses described above. After data collection was completed, a final version of the codes in the codebook was completed, and all 28 interviews were coded based on that codebook in Excel. Data was analyzed using the codebook by trained qualitative research assistants (CS, MF) who conducted the content analysis independently and cross-validated the results by comparing their separate coding sheets. Fifteen interviews were double-coded to ensure accuracy and coding consistency between coders. If there were disagreements between the coding, the coders discussed the differences to achieve consensus. If no agreement could be reached, a third analyst (AT) made the final decision on the coding. A final thematic grouping of the emergent codes was done at the end of the analysis process. Selected quotes are labeled with their Participant ID and type of acupuncture (AA for auricular acupuncture, PA for peripheral acupuncture) received.

## Results

### Description of participants

Between March 2020 and May 2021, 28 qualitative interviews were performed with participants who were enrolled in the acupuncture treatment arms in the parent study between February 2020 and March 2021, with interviews taking place 33-93 days (median 63 days) after ED acupuncture treatment. Participant demographics and characteristics are shown in Table 1. The median age of participants was 44 years, 46.4% were female, and the majority (61%) reported they had never received acupuncture treatment prior to this study. 42% of participants received acupuncture for lower back pain, followed by neck and upper back pain (25%). The median interview length was 30 minutes (range 16 to 47 minutes).

**Table 1:** Participant demographics (N = 28).

Sex		Current employment status	
Male	14 (50%)	Employed Full-Time	16 (57%)
Female	13 (46%)	Unemployed or laid off	4 (14%)
Prefer not to answer	1 (4%)	Retired	6 (21%)
**Age**		Unable to work	2 (7%)
Median (min, max)	44 (20, 79)	**Approximate annual household income**	
**Ethnicity**		Less than $20,000	5 (18%)
Not Hispanic or Latino	26 (93%)	$20,000 to $50,000	7 (25%)
Hispanic or Latino	2 (7%)	$50,000 to $90, 000	7 (25%)
**Race**		Greater than $90,000	2 (7%)
Black or African American	9 (32%)	Prefer not to answer	7 (25%)
White or Caucasian	15 (54%)	**Health Insurance** *(not mutually exclusive)*	
Other or more than one race	3 (11%)	Medicare	8 (29%)
Prefer not to answer	1 (4%)	Medicaid	2 (7%)
**Marital status**		Private	17 (61%)
Never married	12 (43%)	Disability	1 (4%)
Married	11 (39%)	None	4 (14%)
Living together	1 (4%)	Prefer not to answer	1 (4%)
Widowed/Widower	3 (11%)	**Type of Acupuncture received**	
Separated or Divorced	1 (4%)	Auricular Acupuncture	12 (43%)
**Level of Education completed**		Peripheral Acupuncture	16 (57%)
Graduated High school or GED	6 (21%)	**Pain Location**	
Some college	7 (25%)	Neck/ Upper back	7 (25%)
Graduated college	8 (29%)	Lower Back	12 (43%)
Some post-graduate coursework	3 (11%)	Arm	4 (14%)
Completed post-graduate degree	4 (14%)	Leg	5 (18%)
**Prior acupuncture treatment**			
Yes	6 (21%)		
No	17 (61%)		
Missing	5 (18%)		

### Thematic overview

**Fig 1:**
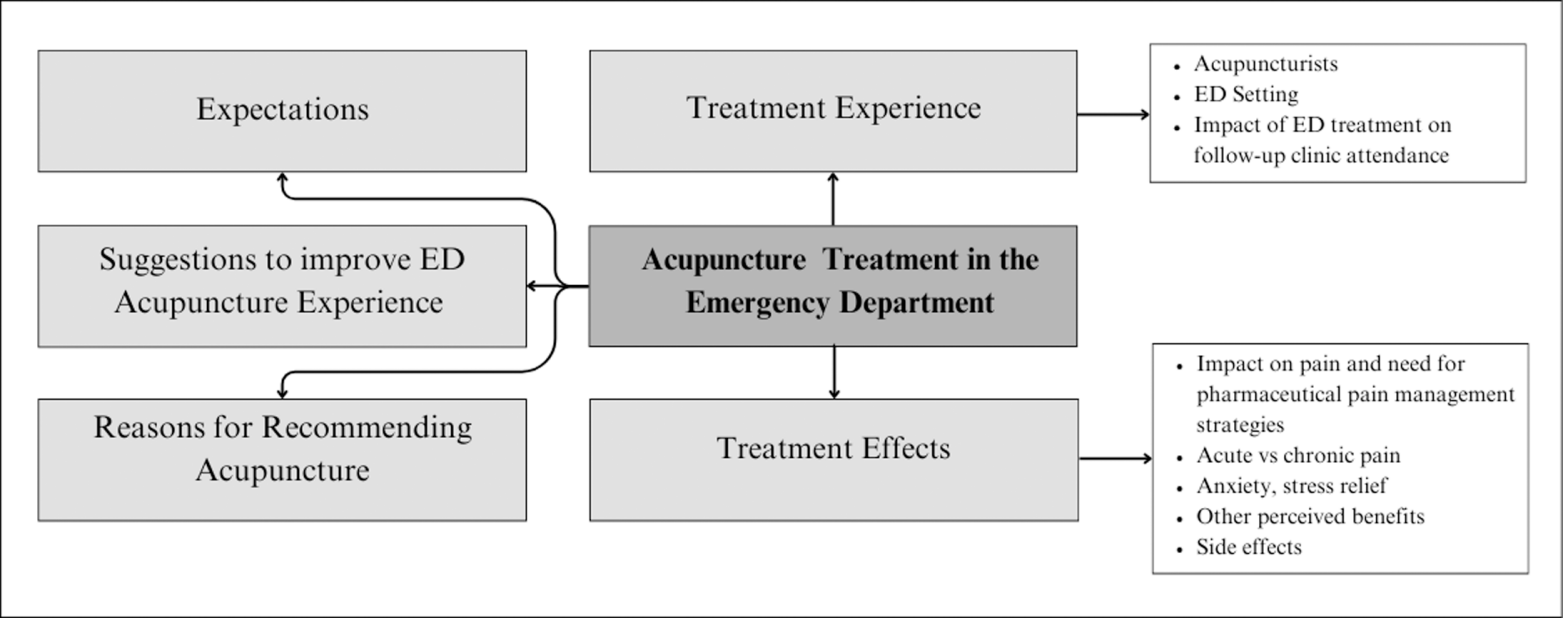
Thematic overview. Emergent themes and codes.

The current analysis focuses on the experiences of patients receiving acupuncture as part of the parent clinical trial while in the ED. Five main themes were identified: (1) expectations and reasons for joining the study, (2) acupuncture treatment experience, (3) reasons for recommending acupuncture, and (4) suggestions to improve the ED acupuncture treatment experience (Fig 1).

Of note, the interviews also covered the treatment experience during the participants’ follow-up visits for outpatient acupuncture treatment at an integrative medicine clinic and experience participating in research. While we are aiming to focus only on the ED-specific acupuncture-related experiences, some participant responses reflected their overall impressions of the follow-up visits and participation in a research study. Thematic mapping with codes and representative quotes for all study participants are shown in Table 2.

**Table 2:** Thematic mapping with exemplary quotations.

Them	Codes	Exemplary quote(s)
**Expectations**	Expectations prior to study and reasons for joining	**050PA** [..] But I expected some pain relief hopefully and just some relaxation from what little I knew about acupuncture. **012PA:** [..] My expectations for it is how effective acupuncture might be for me. [..] if it didn’t do anything, it would still be a plus in terms of my finding out how acupuncture is taking place now and the relative success. **109PA**: I really didn’t know what to think then. I was just interested in some type of alternative medicine, honestly. **113AA**: I didn’t want to go to a doctor and get prescription medicine because that’s just like if you hurt yourself you can put a band-aid on it like you hurt your skin, but after the band-aid comes off you think about it, like I don’t know it’s ever gonna happen again. […] It’s like I feel if it’s working temporarily but as far as permanently I think acupuncture is more lasting.
**Treatment experience**	Acupuncturists	**175PA:** [The acupuncturist] who administered it really knows her stuff, so it went quickly [..]. **217AA**: And I was actually inspired to learn a little bit more about acupuncture, and just the flow of energy throughout the body, and how that can just affect your quality of daily life. **105PA:** And every single thing that I asked,[the acupuncturists] had an answer for. So, I was very happy with that. That kinda made me feel like they knew what they were doing. **132AA**: She was just real well–trained and was just doing it all. She told me the name of the type of treatment she was doing and everything.
	ED Setting	**152PA:** Oh. The privacy was fine. We just drew the curtain and that was fine. [..] As for the noise, I was in too much pain to notice any noise. **028AA:** For the acupuncture, the room was very nice. It was very quiet, a long way from the check in area, and it was nice. **035AA:** Oh, I was in the triage area in the emergency room. It was done like that. It was not comfortable. **068PA:** The room was a little noisy; I don’t know it seemed like an air vent or something- **161PA:** emergency department is too much over there
	Impact of ED treatment on follow-up clinic attendance	**107PA:** It positively made me say, “Okay, I’m going to check this out.” Because it – For what it did for me in the emergency room, I knew, yes, this is something I’m going to pursue. **097PA:** Well, I knew I was in good hands because, as I said, [..] explained things and reduced my anxiety. **175PA:** Yes, I did because I guess as a result of the initial treatment in the ER, of course, because it worked. I suppose if it hadn’t worked, I might not have signed up.
**Treatment effects**	Impact on pain and need for pharmaceutical pain management strategies	**075PA:** I realized that the acupuncture was actually more healing than being drugged up. **071PA:** Every day I would walk in there, I would be in pain. And when I leave, I wouldn’t be in pain. And I hadn’t had not one drop of medication. And it was just baffling to me. **092PA:** Oh, it met my expectations. There’s no doubt about it. and I’ve been able to continue with just extra strength Tylenol. I haven’t felt the need to go to anything stronger at all. **068PA:** Yeah. I saw immediate improvement when I started in the emergency department. When she put the one in my lip, there was a certain pain in my back that just disappeared automatically
	Acute vs chronic pain	**019PA:** I couldn’t really tell because I don’t have chronic pain, so I couldn’t really tell if it was having any kind of effect at all. For me, I don’t know if I was the right kind of patient to do this study on because I had some acute pain with my foot [..]. **138AA:** Oh yes. Even the day… well, I had a headache, and it was just gone after the treatment, yes. **028AA:** I can’t expect that great results on a short period of time frame. **018AA**: I guess the whole point of this study was to be able to reduce pain while in an emergency type situation, and it didn’t do that.
	Anxiety, stress relief	**097PA**: [..] the treatment in the emergency room helped me calm down. And it wasn’t so much a pain reliever as it was a calming effect. **050PA:** I feel like I’m quicker to feel anxious and tense. And it was just sort of like that kind of – acupuncture I feel like kind of muted that for me. **134AA:** It does help me with my frustration sometimes because I was getting upset about the situation that was going on but […] the treatments helped me out and that’s why I say it just improved on my personality
	Other perceived benefits	**092PA:** I wasn’t sleeping that well. And of course, I’ve just been through pretty aggressive treatment for bone cancer. But this relaxed me to a point I’ve started getting real good sleep. I sleep right through the night. **217AA:** Just kind of keep maintenance of [..] the energy flow throughout my body. So, yeah, that’s something I will look forward to even if I’m not necessarily in pain.
	Side effects	**092PA:** Battlefield [auricular] or something, […] I found that very uncomfortable. **017AA:** I found that I had the ear kind, it actually turned out, in the end, to be painful to me. **117AA**: I was sore some. **130PA:** Mild discomfort, yeah, mild to none I’d say. It was probably I think there was one time when there was a needle near my thumb or in that area, kinda near my thumb, and me moving my finger instead of being still kind of made me feel uncomfortable,
**Reasons for recommending acupuncture**	Reasons for recommending acupuncture	**050PA:** [..] I don’t know if it was relieving tension that then helped some of the pain or – but I definitely noticed an improvement in my pain. And just an improvement in anxiety and stress. So, I feel like it was treating multiple problems and that’s good. **149AA:** Yeah because a lot of times, even my wife, I – as of right now, she’s saying that she want a better life than feeling the chronic pain that she’s having. And right now, her doctor is not fulfilling her needs and her pain management. **077AA**: Yes, I would, especially if I had a friend that have muscle spasms like I do every now and again. I would advise them to try it.
**Suggestions to improve ED acupuncture experience**	Suggestions to improve ED acupuncture experience	**071PA:** How big the needles were. I’m deathly afraid of needles, which is really weird because I’m diabetic. A finger prick is different than little needles everywhere. [..] **012PA:** Okay well, I guess for me; personally, it would be, I guess, a little bit more of the science of the acupuncture. **167PA:** think using a few more acupuncture needles probably would have helped me out. […] It would probably be a lot stronger.

#### 1: Expectations.

1.1. Expectations prior to study and reasons for joining: Expectations for the study and the reason to join were often the same or intertwined for participants. The most reported reason for joining the study and expectations for the acupuncture effect prior to the study was hoping it ”*might offer some pain relief” (017AA),* followed by hoping to experience relaxation and anxiety relief. The majority of participants had not received acupuncture treatment before, and were curious to find out if it could be a beneficial complementary treatment to decrease their pain. They were interested in joining because either they had heard about positive experiences from people they knew or they did not know what to expect at all. This curiosity of *“[…] finding out how acupuncture is taking place […]” (012PA)* and willingness to participate was especially true in those for whom medications had not brought the desired results and in those who had tried to reduce their medication dosage.

*“Really, out of interest. I’d rather do anything else that I can rather than take medicine for pain management. So, I thought it might be interesting, and it was.” (109PA)*

Other reasons for joining were the ability to receive acupuncture treatment at no cost, the convenience of receiving the treatment while in the ED, and less time spent in the ED waiting room as they were taken to a separate private ED space for study activities and acupuncture treatment while waiting to be assigned a room. In addition, some participated because they were curious about being a participant in a clinical study and hoped to contribute to knowledge generation that could improve clinical care in the future.

#### 2: Treatment experience.

Factors that influenced both positive and challenging experiences while receiving acupuncture treatment in the ED can be grouped into the interactions and information provided by the acupuncturists, which were predominantly positive, the ED setting, which elicited more mixed responses about its convenience versus noise and privacy levels, and the impact having received acupuncture during their ED visit had on continuing treatment in the outpatient setting.

2.1. Acupuncturists: The overall experiences with the acupuncturists were markedly positive. The majority of participants reported feeling comfortable with the acupuncturists, and perceived them as well-trained, professional, knowledgeable (someone who *“really knows her stuff” (175PA),* kind, helpful, open, competent, establishing trust with the participants, providing adequate explanations, and contributing to a calming experience overall.

*“So, everybody was, I mean I give them 100%. If I was a millionaire, I would give everybody a tip. That’s how I felt.” (134AA)*

The high level of responsiveness and communication about what to expect before and throughout the treatment procedures was considered important for making the patients feel comfortable. This included specifics about needle placements such as “*these are ‘the points’ what are being focused on, and this is the side effect I should be expecting”* (149AA) and length of treatment. Another key aspect was how effectively acupuncturists handled side effects, follow-up questions, or concerns. The acupuncturists’ affiliation with the academic center and participation as clinicians in a research study further built trust in their competence. However, one participant pointed out that it would have been helpful to receive more information on the acupuncturist’s prior training:

*“But I think overall maybe upfront saying even if it’s just a bio and an email of, ‘Our acupuncturists all have been certified by XYZ,’ or, ‘have this kind of training,’ type thing.” (130PA)*

2.2. ED Setting: Generally, the ED was considered a comfortable environment and atmosphere, with sufficient privacy and confidentiality maintained despite the *‘regular ED noise’ (109PA)*. However, the perceived comfort level varied with some of the specific private room spaces (e.g., rooms next to loud equipment were less comfortable) and the type of treatment chairs or beds. The duration of acupuncture treatment and study participation in the ED was generally deemed acceptable, although one person who reported an onset of lightheadedness perceived the treatment as a bit too long.

While the fast-paced environment in the ED and with the initial study invitation invoked feelings of being rushed for two participants, the ED setting did not appear to compromise the experience or perceived privacy for most participants. In fact, participants appreciated being taken out of the waiting room to participate in the study. They also liked receiving the treatment prior to being seen by their doctors, as it facilitated their pain relief sooner. However, one participant raised the concern that if acupuncture does not give immediate pain relief in the ED, people may not think it is an appropriate ED treatment:

*“Opioid addiction and all that stuff is really bad. So, it’d be a great thing if you could come up with a way to not have to give people pain medication. But I just don’t, the problem is, is if it’s not 100% effective, […] in certain situations it’s not gonna work.” (018AA)*

Some participants expressed muted enthusiasm for their initial acupuncture treatment due to stressors and frustrations related to delays in care that are common in the ED setting, but most participants had positive remarks regarding the convenience of receiving the treatment directly in the ED because ‘*most people are not able to come to a [….] different location’ (167PA)* led them to support offering acupuncture in the ED setting.

Participants noted that integrating the acupuncture treatment into the ongoing medical assessments and standard care provided in the ED could be challenging due to conflicting medical procedures (e.g., taken to X-ray), as well as potential delays in usual medical care due to receiving acupuncture treatment.

2.3. Impact of ED treatment experience on follow-up clinic attendance: Motivating factors to continue acupuncture treatment in the clinic setting after ED treatment included immediate pain relief while in the ED, or in the cases where the initial treatment was not as effective as they had hoped for, giving acupuncture *‘a chance to work’* (117), good communication and interactions with the study team, and having the option to receive ongoing acupuncture free of cost.

While the initial treatment experience in the ED positively influenced most participants’ decision to attend acupuncture follow-up visits, one participant noted that for them, the initial ED experience felt rushed. They state that this could raise concerns that the follow-up visits would be similarly rushed, possibly negatively influencing the follow-up adherence, stating that *“besides […] the relief of the pain and stuff, it felt like a rushed experience, and that gave me pause” (*130PA).

#### 3: Treatment effects.

Overall, the vast majority of participants reported some positive treatment effect such as pain relief, decreased usage of pharmaceutical pain management strategies, anxiety or stress relief, and improved sleep. While for some participants the treatment did not yield the expected results, we could not identify any patterns between expectancy of positive outcomes and reported positive treatment effects.

3.1. Impact of acupuncture on pain and need for pharmaceutical pain management strategies: The majority of participants reported a decrease in their pain levels, some to the point *that ‘acupuncture was actually more healing than being drugged up’* (075) and were fully pain-free after acupuncture treatment. One participant even reported being able to stop taking stronger pain medication and muscle relaxants and reduce their frequency of other pain medications throughout the day due to acupuncture, while another was able to fully refrain from taking pain medications and experienced complete pain relief with acupuncture treatment:

*“[..] It just opened up my mind to a whole new way of not having to take oral medication to relieve pain. …[Before acupuncture treatment], I would be in pain. And when I leave [after treatment], I wouldn’t be in pain. And I hadn’t had not one drop of medication. And it was just baffling to me.”* (071PA)

Two of the participants expected to experience a faster onset of pain relief. Yet, both reported to eventually achieve a more continuous and longer lasting pain relief with acupuncture treatment, compared to other pain management strategies such as injections.

Other participants felt that it was difficult to ascertain whether their pain relief was due to acupuncture or to the additional intervention strategies that they were utilizing concurrently, or whether the effect was due to personal expectations including *‘something that [they were] so desperately trying to convince [themselves] of emotionally*’ (017AA).

For some participants, acupuncture *‘was what [they] expected and it worked’* (097PA), but it didn’t fully resolve their pain, requiring supplemental treatment modalities such as physical therapy. Only two people reported no decrease in pain at all, stating that the *‘actual treatment itself did not help’* (117AA) and that they ‘*didn’t really feel any different’* (018AA) and would therefore not continue acupuncture to treat their pain, while another person experienced an increase in pain after receiving auricular acupuncture (17AA). Two additional participants reported no immediate results after the ED acupuncture treatment.

*3.1.1. Acute vs chronic pain*. For some participants with underlying chronic pain, the perceived benefit of acupuncture for acute pain was deemed not as strong as for chronic pain, wondering how much of the improvements could be attributed to the natural healing process after an acute injury. One participant reported they were planning to continue using acupuncture for chronic pain, but would not use it for acute pain resulting from an injury:

*“I might [use acupuncture] in a circumstance where I do have chronic pain. Right now, I don’t. There had been a couple times where I was in chronic pain. I had a hip injury and really there wasn’t a whole lot of things that would help.” (019PA)*

3.2. Anxiety and stress relief: In addition to meeting their expectations for pain relief, some participants additionally pointed out the unexpected benefits of acupuncture being *‘very good for stress and anxiety’*(217AA) and *‘learn[ing] how to relax, […] how to take it easy’*(161PA). In particular, for some the initial treatment in the ED had more of a positive calming effect than the anticipated pain relief, which was frequently unexpected and led some participants to suggest offering acupuncture to anxious patients in the ED prior to receiving any other medical care. Other participants reported that being able to share the healing experience with the acupuncturist created a positive psychological impact.

3.3. Other perceived benefits: Additionally, having a treatment experience that wasn’t ‘*run of the mill’* (097PA) created a different but positive experience for some. Additional benefits included learning more about energy flow in the body and gaining a deeper understanding of acupuncture and its response on the physical body. One participant reported improved sleep quality*: ‘But this relaxed me to a point I’ve started getting real good sleep. I sleep right through the night.’* (092PA)

3.4. Side effects: The vast majority of participants tolerated the treatments very well and reported no negative side effects. Of those who did experience side effects, the most common was mild pain or discomfort with the needle placements, more commonly with auricular acupuncture ear needles that usually resolved during the treatment session or *‘later that day’* (117AA). Participants receiving auricular acupuncture cited specific adverse effects, with one person asking to discontinue the auricular approach due to pain and another reporting a skin reaction to the press needle adhesive.

Others reported delayed effects including mild soreness or mild bruising a day after the treatment. Less common reactions included muscle twitching in the legs. In addition, one participant reported no discomfort during the treatment but later experienced tingling in the feet and a sensation of heaviness in the legs *‘like they were made of cement’* (028AA) which self-resolved, and another reported leg numbness with prolonged ambulation that would resolve when sitting down to rest (071PA). However, both participants noted that these latter effects could be attributed to their underlying health issues and overall state-of-being such as being dehydrated or in a *‘different train of mind’,* rather than due to acupuncture (035AA).

#### 4: Reasons for recommending acupuncture.

Overall, participants voiced that they would or already had recommended acupuncture to friends and family based on their own positive experiences, most predominantly for pain relief but also to reduce stress. One participant specifically recommended including acupuncture as an integral part of ED treatment prior to seeing physicians, as a way to reduce stress and create a more calming atmosphere (217AA). Those who did not personally have a positive treatment effect were less likely to recommend it.

When recommending acupuncture to friends and family, several participants mentioned the importance of reducing the fear around needles as an obstacle to seeking acupuncture treatment:

*“I know a lot of people are scared of needles, but I’d advise them to try it one time. And I think they’ll like it like I did. I think they’ll continue to go to the appointments like I did.”(077AA)*

#### 5: Suggestions to improve ED acupuncture experience.

Overall, participants reported a positive experience participating in acupuncture treatments, and most could not think of ways to improve the overall experience.

Among those with suggestions for improving the experience in the ED, participants pointed out that at times it seemed like there were delays due to searching for supplies, so sufficient acupuncture supplies such as needles would need to be readily available and easy to access in the treatment rooms. Some also cited the importance of creating a more comfortable and quiet treatment environment, as well as greater privacy during the ED visits. In addition, some participants suggested providing more initial information and details about acupuncture and how it can serve as a promising alternative for pain management, the locations and rationale for needle placements during treatment, and testimonials from previous participants that address potential concerns and fears about acupuncture, *‘more so just people saying that the treatment was beneficial for them’* (217AA). One participant also suggested including and instructing on *‘some sort of exercise to help the condition. To add and enhance the treatment’* (012PA).

## Limitations

This qualitative study recruited participants from the initial pilot phase of a randomized clinical trial of acupuncture, so this population may differ from the general ED population in being more open to acupuncture and thus more inclined to positive feedback. Additionally, not everybody who was contacted agreed to participate in the interviews. Both circumstances may lead to a selection bias among those who did participate in the acupuncture trial and who agreed to participate in the interviews, as having more positive or more negative experiences than the general study population. However, the qualitative findings reported in this study are in line with the favorable quantitative acceptability survey results from the parent study, which had a much higher response rate.[16]

## Discussion

### Summary of main findings

The purpose of this qualitative study was to explore patient experiences of acupuncture in an ED setting and identify strategies to improve those experiences. Overall, ED patients with acute musculoskeletal pain expressed interest in participating in an acupuncture study due to a desire for pain relief and, to a lesser extent, anxiety and stress relief. Many cited the novelty of acupuncture compared to medications and the convenience of receiving it during their ED visit, at no additional cost, as reasons for participating. Communication was key to patient engagement, with clear and comprehensive explanations of the acupuncture treatment process, study-related activities, and attention to patient concerns and questions being important. Most patients wanted a basic understanding of how acupuncture works, what the needles are like and how they are used during treatment.

Most participants reported an overall positive treatment experience with acupuncture, particularly if they experienced pain relief. While there was variability in the treatment response, most reported that acupuncture met their expectations for pain improvements and many reported additional positive impacts on stress, anxiety, depression and sleep quality. Only two participants reported no improvement. Acupuncture was overall tolerated well with few participants reporting side effects, with the most common being mild pain or discomfort at some of the needle insertion sites, particularly the ears. Establishing trust and a therapeutic relationship with acupuncturists who are open, friendly, communicative, and conveying expertise were also important. The ED environment, despite being busy, noisy and stressful, was perceived as sufficiently comfortable, relaxing and private for acupuncture treatment. Overall, participants who had a positive experience with acupuncture in the ED were more likely to recommend acupuncture to others. Our findings suggest that acupuncture has the potential to be more widely integrated into emergency departments to provide a nonpharmacologic treatment option. However, additional time-cost-effectiveness evaluations are needed to assess its practical feasibility as a standard-of-care treatment option.

### Comparison of ED acupuncture experience with existing literature

Our findings are in line with existing literature showing similar high levels of interest and acceptability of acupuncture among patients in the ED setting. Two cross-sectional studies of ED patients with pain showed that most patients are willing to try nonpharmacologic treatments including acupuncture for their pain.[21] A few observational studies showed that most ED providers and ED patients presenting with pain, anxiety and nausea found acupuncture treatment delivered in the ED to be acceptable.[22,23] Most of these patients reported symptom relief, high satisfaction scores, and low side effects with acupuncture treatment. Similar efficacy, low side effects, and satisfaction with acupuncture were reported in a recent systematic review of randomized trials of acupuncture delivered in the ED.[24]

To our knowledge, this is the first study to explore experiences of patients receiving acupuncture in an ED setting. However, similar themes to our results were found in other qualitative studies investigating the experiences of patients receiving acupuncture in clinic settings including those attending low-cost community-based acupuncture clinics.[25–28] These studies found that participants with a specific need for treatment, such as poorly controlled pain, wanted to try something new besides medications even if they did not “believe” in it prior to trying it. In addition, factors that enhanced patients’ willingness to try and their overall experience with acupuncture included open and compassionate communication from the acupuncturist, therapeutic alliance with their acupuncturist, increasing their understanding of and familiarity with acupuncture including its safety and low side effect profile and existence as a millennia-old treatment, and holistic outcomes including improvements in pain, anxiety and sense of well-being as well as other positive effects. Important reported barriers to use of acupuncture included high cost and limited access to treatment in terms of both time and location availabilities. One study of acupuncture clinical trial participants showed that participants were more likely to attend treatment sessions if they felt the treatment was effective for them and that appointments were accessible and convenient, particularly with regard to schedule and travel.[26]

### Using the qualitative results to improve ED acupuncture experience

Participants identified elements of the recruitment process, treatment experience and overall treatment effects during the parent trial that were beneficial to their overall experience as well as areas for improvement that could be applied to the concurrent clinical trial. Based on participant feedback, recruitment materials were updated to include simple but more detailed explanations of what acupuncture entails, including pictures of the hair-thin needles to alleviate the fear of needles expressed by many, brief description of how acupuncture works including stimulation of acupoints, brief summary of current understanding and data on acupuncture, brief biographical information and credentials of study acupuncturists and research team, identification of alternative quieter private ED spaces (e.g., unused treatment and consultation spaces) for waiting room patients to receive study information and acupuncture, and use of acupuncture satisfaction surveys to give individual acupuncturists feedback on patient-reported experiences and satisfaction with their interactions. Finally, the study team engaged in ED provider and staff talks as well as several opportunities to personally experience acupuncture treatment in order to improve ED clinical personnel understanding of, engagement with, and support of acupuncture treatment in the ED.

In conclusion, ED patients with musculoskeletal pain were interested in receiving acupuncture treatment for their pain while in the ED, derived added benefits, including treatment of anxiety and a therapeutic alliance with acupuncturists, and reported high satisfaction with and acceptability of the experience. Including qualitative study components in clinical trial designs to receive participant feedback on their experiences could be used to update specific details of recruitment materials and acupuncture delivery within the ED setting to improve both individual patient experiences and overall study performance and inform future research priorities.

## Supporting information

S1 Table Research Team Characteristics. (PDF)
