# Supplementary material for: Participant experiences receiving acupuncture for acute musculoskeletal pain in an emergency department: A qualitative evaluation
Source: PLoS One. 2025 Feb 12;20(2):e0318345. doi: 10.1371/journal.pone.0318345 (PMC11819596; doi:10.1371/journal.pone.0318345)
Supplement: S1 Table — (PDF) [file pone.0318345.s001.pdf]

**S1 Table.** Research Team Characteristics

| Initials | Role | Credentials | Occupation | Gender | Experience and Training | Relationship with Participants |
|---|---|---|---|---|---|---|
| OCT | Participant recruitment, Interviewer, Codebook developer | BA | Clinical Research Coordinator | Female | Experience with qualitative and quantitative research methods | Relationship established at time of consent. Participants did not have any information about the researcher outside of the researcher's role on the study team. |
| ERW | Participant recruitment, Interviewer | MHA | Clinical Research Coordinator | Female | Experience with qualitative, quantitative research methods, study coordination, and data management. | Relationship established at time of consent. Participants did not have any information about the researcher outside of the researcher's role on the study team. |
| MF | Coder and Codebook developer | BS | Clinical Research Coordinator | Female | Experience with qualitative and quantitative research methods | No direct participant interactions |
| CS | Coder and Codebook developer | BA | Research Assistant | Female | Experience with qualitative and quantitative research methods | No direct participant interactions |
| SE | Study supervision, Coding input, Codebook developer | MD, PhD | Faculty | Female | Experience with qualitative and quantitative research methods | Principal investigator |
| AT | Coder, Codebook developer, supervision, Data management | DPT, MSc | Postdoctoral Associate | Female | Extensive didactic and experiential training in qualitative research methods | No direct participant interactions |
| AEO | Data management, Study coordination | MPH | Clinical Research Coordinator | Female | Experience with qualitative and quantitative research methods | No direct participant interactions |
